# Anomaly Detection in Paleoclimate Records Using Permutation Entropy

**DOI:** 10.3390/e20120931

**Published:** 2018-12-05

**Authors:** Joshua Garland, Tyler R. Jones, Michael Neuder, Valerie Morris, James W. C. White, Elizabeth Bradley

**Affiliations:** 1 Santa Fe Institute, 1399 Hyde Park Rd., Santa Fe, NM 87501, USA; 2 Institute for Arctic and Alpine Research, University of Colorado Boulder, Boulder, CO 80309, USA; 3 Department of Computer Science, University of Colorado Boulder, Boulder, CO 80309, USA

**Keywords:** paleoclimate, permutation entropy, ice core, anomaly detection

## Abstract

Permutation entropy techniques can be useful for identifying anomalies in paleoclimate data records, including noise, outliers, and post-processing issues. We demonstrate this using weighted and unweighted permutation entropy with water-isotope records containing data from a deep polar ice core. In one region of these isotope records, our previous calculations (See Garland et al. 2018) revealed an abrupt change in the complexity of the traces: specifically, in the amount of *new* information that appeared at every time step. We conjectured that this effect was due to noise introduced by an older laboratory instrument. In this paper, we validate that conjecture by reanalyzing a section of the ice core using a more advanced version of the laboratory instrument. The anomalous noise levels are absent from the permutation entropy traces of the new data. In other sections of the core, we show that permutation entropy techniques can be used to identify anomalies in the data that are not associated with climatic or glaciological processes, but rather effects occurring during field work, laboratory analysis, or data post-processing. These examples make it clear that permutation entropy is a useful forensic tool for identifying sections of data that require targeted reanalysis—and can even be useful for guiding that analysis.

## 1. Introduction

Paleoclimate records, such as ice and sediment cores, provide us with long and detailed accounts of Earth’s ancient climate system. Collection of these data sets can be very expensive, and the extraction of proxy data from them is often time consuming, as well as susceptible to both human and machine error. Ensuring the accuracy of these data is as challenging as it is important. Most of these cores are only fully measured one time, and most are unique in the time period and region that they “observe”, making comparisons and statistical tests impossible. Moreover, these records may be subject to many different effects—known, unknown, and conjectured—between deposition and collection. These challenges make it very difficult to understand how much information is actually present in these records, how to extract it in a meaningful way, and how best to use it while not overusing it.

An important first step in that direction would be to identify where the information in a proxy record appears to be missing, disturbed, or otherwise unusual. This knowledge could be used to flag segments of these data sets that warrant further study, either to isolate and repair any problems or, more excitingly, to identify hidden climate signals. Anomaly detection is a particularly thorny problem in paleorecords, though. Collecting an ice core from polar regions can cost tens of millions of $US. Given the need for broad geographic sampling in a resource-constrained environment, replicate cores from nearby areas have been rare. This has restricted anomaly detection methods in paleoscience to the most simple approaches: e.g., discarding observations that lie beyond five standard deviations from the mean. Laboratory technology is another issue. Until recently, for instance, water isotopes in ice cores could only be measured at multi-centimeter resolution, a spacing that would lump years or even decades worth of climate information into each data point. The absence of ground truth is a final challenge, particularly since the paleoclimate evidence in a core can be obfuscated by natural processes: material in a sediment core may be swept away by an ocean current, for instance, and ice can be deformed by the flow of the ice sheet. These kinds of effects can not only destroy data, but also alter it in ways that can create spurious signals that appear to carry scientific meaning but are in fact meaningless to the climate record.

Thanks to new projects and advances in laboratory techniques, the resolution challenge is quickly becoming a thing of the past [1]. In addition to the recently measured West Antarctic Ice Sheet (WAIS) Divide ice core, many additional high-resolution records are becoming available, such as the South Pole Ice Core (SPC) [2] and the Eastern Greenland Ice Core Project (EGRIP) [3]. Replicate data is on the horizon as well, which may solve some of the statistical challenges. The SPC project, for instance, will involve dual analysis (i.e., two replicate sticks of ice) from three separate subsections of the ice core. However, replicate analyses of deep ice cores beyond a few hundred meters of ice will not occur any time soon, let alone multiple cores from a single location (see Endnote [4]—which refers to References [5,6]). Thus, a rigorous statistics-based treatment of this problem is still a distant prospect for deep ice cores. In the meantime, information-theoretic methods—which can work effectively with a *single* time-series data set—can be very useful. In previous work, we showed that estimates of the Shannon entropy rate can extract new scientific knowledge from individual ice-core records [7,8]. There were hints in those results that this family of techniques could be more generally useful in anomaly detection. This paper is a deeper exploration of that matter.

To this end, we used permutation entropy techniques to study the water-isotope records in the WAIS Divide ice core. The resolution of these data, which were measured using state-of-the-art laboratory technology [1,9], is an order of magnitude higher than that of traditionally measured deep ice core paleoclimate records (e.g., [10]) and also representative of new data sets that are becoming available. We identified abrupt changes in the complexity of these isotope records using sliding-window calculations of the permutation entropy [11] (PE) and a weighted variant of that method known as weighted permutation entropy (WPE) that is intended to balance noise levels and the scale of trends in the data [12]. Via close examination of both the data and the laboratory records, we mapped the majority of these abrupt changes to regions of missing data and instrument error. Guided by that mapping, we remeasured and reanalyzed one of these segments of the core, where the PE and WPE results suggested an increased noise level, and the laboratory records indicated that the processing had been performed by an older version of the analysis pipeline. The PE and the WPE of this newly measured data—produced using state-of-the-art equipment—are much lower and consistent with the values in neighboring regions of the core. This not only validates our conjecture that permutation entropy techniques can be used to identify anomalies but also suggests a general approach for improving paleoclimate data sets in a targeted, cost-effective way.

Permutation entropy is a complexity measure: it reports the amount of new information that appears, on average, at each point in a sequence. In the context of a time series, this translates to a measure of how information propagates forward temporally. This has implications for predictability [13,14], among other things; indeed, these measures have been shown to converge to the Kolmogorov–Sinai entropy under suitable conditions [15,16], as described in Section 2.2. The goal here is not to measure the complexity of paleoclimate data in any formal way, however. Our intent, and the underlying conjecture behind our approach, are more practical: abrupt changes in a quantity like permutation entropy suggest that something changed, either in the system or the data. The following section describes the data and methods that we used to demonstrate the efficacy of that approach.

## 2. Materials and Methods

### 2.1. Data

**Figure 1:**
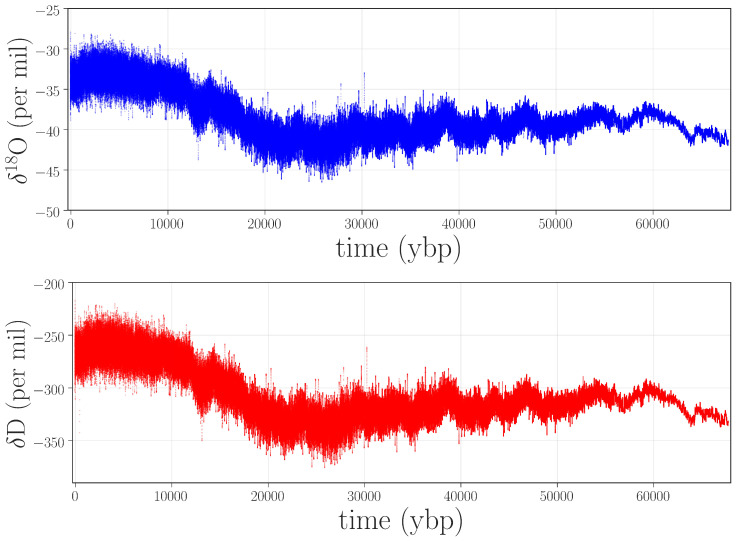
Water-isotope records for $\delta^{18}$O (top panel) and δD (bottom panel) taken from the West Antarctic Ice Sheet (WAIS) Divide ice core (WDC).

As a proof of concept for the claim that information theory can be useful in detecting anomalies in paleorecords, we focused on water-isotope data from the West Antarctic Ice Sheet (WAIS) Divide ice core (termed WDC hereafter) [9]. These data, which consist of laser absorption spectroscopy measurements of the isotopic composition of the ice, are proxies for local temperature and regional atmospheric circulation during the past 67,000 years. The specific variables that we consider are the ratios of the heavy and light isotopes of hydrogen (${}^{2}$H/${}^{1}$H) and oxygen (${}^{18}$O${/}^{16}$O). Time-series traces of these ratios, which we will identify as δD and $\delta^{18}$O in the rest of this paper, are shown in Figure 1.

The details of the experiment and data processing are as follows. The water-isotope data were recorded at a rate of 1.18 Hz (0.85 s intervals). Ice samples were moved through a cavity ring-down spectroscopy continuous flow analysis (CRDS-CFA) system [1] at a rate of 2.5 cm/min, yielding millimeter resolution. Registration of the laser that tracks the depth along the core can be a challenge, particularly in the brittle zone, a section of ice from approximately 577–1300 m (≈2.34–6 kybp) in the WDC that tends to break apart when the overburden pressure of the ice sheet is removed, allowing air bubbles to expand and shatter the core. This issue will return later in this paper. The data were then averaged over non-overlapping 0.5 cm bins. The ratio of heavy to light water isotopes in a water sample is expressed in delta notation [17,18] relative to Vienna Standard Mean Ocean Water, where VSMOW has been set to 0‰ by the International Atomic Energy Agency (IAEA): $\delta =1000(R_{sample}/R_{VSMOW}-1)$, where *R* is the isotopic ratio. The $\delta^{18}$O and δD symbols refer to fractional deviations from VSMOW, normally expressed in parts per thousand (per mille or ‰).

Converting the δD and $\delta^{18}$O data from the depth scale to an age scale is a major undertaking, as well as a significant source of uncertainty. Constructing the age–depth model for the WAIS Divide core required dozens of person-months of effort. In the upper ≈1500 m of the core—the past 31,000 years—the age–depth relationship for the WDC water isotope record was determined by combining annual dating of high-resolution (<1 cm) measurements of electrical conductivity, dielectrical properties, and sulfur, sodium, and black carbon concentrations [19]. Below that, the age–depth curve relies on tie points—known events, like volcanic eruptions, that leave synchronizable signals in different climate records—to the Hulu Cave timescale [20], with modeling and smoothing used to fill in the gaps between those points. This is common practice in age models for deep ice cores, where compression and diffusion (see Endnote [21]—which refers to References [1,22]) eradicate the annual variations below a certain depth. Similar issues arise in age models for sediment cores, where marine organisms mix the material from different time periods. These and other effects that deform the timelines (e.g., brittle zone deformities introduced into the depth scale during the registration process) are critically important considerations when one is doing any kind of time-series analysis on paleoclimate data sets.

The final stage in the data-processing pipeline addresses the uneven temporal spacing of the samples. Because of compression, 0.5 cm of ice—the width of a sample—represents roughly 1/40th of a year of accumulation near the top of the WDC and roughly 1.4 years near the bottom (see Endnote [23]—which refers to Reference [8]). One could certainly calculate PE or WPE on such a sequence, but the timeline of the results would be deformed because of the temporally irregular spacing of the data points. Since we are specifically interested in the timescales of the changes in complexity, we needed to perform our calculations on data that are evenly spaced in time, so we interpolated the δD and $\delta^{18}$O data to an even 1/20th year spacing using a combination of downsampling and linear splines. While this is standard practice in the paleoclimate field, it is not without problems if one plans to use information-theoretic methods on the results, as described in more detail in Section 2.2. Indeed, irregular temporal spacing is a fundamental challenge for any kind of time-series analysis method, and the effects of interpolation methods used to regularize the temporal spacing of these data sets must be considered very carefully if one is doing sophisticated nonlinear statistics on the results. The following section describes how this plays out in the context of WPE calculations on ice cores; several recent papers offer treatments of this matter in more general terms for other kinds of paleoclimate data [24,25,26,27,28].

### 2.2. Complexity Estimation

Permutation entropy (PE)—a measure of how much new information appears at each time step, on average, across the computation window—was originally proposed as a “natural complexity measure for a time series” [11]. Since its conception, PE has been shown to converge to the Kolmogorov–Sinai entropy [15,16] or the metric entropy [29,30] under a variety of conditions and depending on the features (e.g., stationary, ergodic) of the underlying generating process. It has also been shown to correlate with intrinsic predictability in a variety of time series [13,14]. For the purposes of this paper, we simply view permutation entropy as a measure of the temporal complexity of a time series and we treat abrupt changes in that complexity as a signal of possible anomalies, naturally occurring or data related.

Permutation entropy calculations combine traditional information-theoretic probability estimates with ordinal analysis, a process by which time-ordered elements of a time series, also known as delay vectors $\left[x_{t},x_{t+\tau },\ldots ,x_{t+(m-1)\tau }\right]\equiv X_{t}^{m,\tau }$, are mapped to an *ordinal pattern*, or “permutation”, of the same size. If the first, second, and third points in a time series were $X_{1}^{3,1}=\left[x_{1},x_{2},x_{3}\right]=\left[17.5,-1.8,4.1\right]$, for instance, the corresponding permutation would be ϕ([17.5,−1.8,4.1])=231 since $x_{2}\leq x_{3}\leq x_{1}$. We will refer to this mapping as $\phi :R^{m}\to S_{m}$, which accepts an *m*-dimensional delay vector and returns the corresponding permutation π, a member of $S_{m}$, the set of permutations of length *m*. After using ϕ to convert the time-series data into a series of permutations, one computes the permutation entropy using the formula:

(1) $$H\left(m,\tau \right)=-\sum_{\pi \in S_{m}}p\left(\pi \right)\log_{2}\left(\pi \right)$$

where p(π) is the estimated probability of each permutation π occurring in the time series, calculated as follows:

(2) $$p\left(\pi \right)=\frac{\left|\{t|t\leq N-\left(m-1\right)\tau ,\phi \left(X_{t}^{m,\tau }\right)=\pi \}\right|}{N-(m-1)\tau }$$

Here, |·| is set cardinality.

Notice that the mapping ϕ, as defined above, does not distinguish between [17.5,−1.8,4.1] and [17000,−1.8,4000]; both will be mapped to the same 231 permutations. This may be inappropriate if the observational noise is larger than the larger-amplitude trends in the data, or if there is meaningful information in the amplitude of the signal [12]. The now-standard way to address these concerns, *weighted* permutation entropy (WPE) [12], effectively emphasizes permutations that are involved in “large” features and de-emphasizes those whose amplitudes are small relative to the features of the time series. This is accomplished by weighting each permutation by its variance:

(3) $$w\left(X_{t}^{m,\tau }\right)=\frac{1}{m}\sum_{j=1}^{m}{\left(x_{t+(j-1)\tau }-\bar{X_{t}^{m,\tau }}\right)}^{2}$$

where $\bar{X_{t}^{m,\tau }}$ is the arithmetic mean of the values in $X_{t}^{m,\tau }$. The weighted probability of a permutation is defined as:

(4) $$p_{w}\left(\pi \right)=\frac{\sum_{t\leq N-(m-1)\tau }w\left(X_{t}^{m,\tau }\right)\cdot \delta \left(\phi \left(X_{t}^{m,\tau }\right),\pi \right)}{\sum_{t\leq N-(m-1)\tau }w\left(X_{t}^{m,\tau }\right)}$$

where δ(x,y) is 1 if x=y and 0 otherwise. The final calculation of WPE is similar to Equation (1) above, but with the weighted probabilities:

(5) $$H_{w}\left(m,\tau \right)=-\sum_{\pi \in S_{m}}p_{w}\left(\pi \right)\log_{2}p_{w}\left(\pi \right).$$

Following standard practice, we normalize both PE and WPE results by dividing by $\log_{2}\left(m!\right)$, which causes them to range from 0 (low complexity) to 1 (high complexity) [13].

The temporal resolution of a permutation entropy analysis is dictated by the span of the data across which the sum is taken. Since we are interested in the changing patterns in the δD and $\delta^{18}$O traces described at the end of Section 2.1—not overall results that are aggregated across the entire traces—the calculations in this paper employ Equations (1) and (5) in sliding windows across those traces. Choosing the size *W* for those sliding windows is not trivial. The minimum window width is effectively dictated by the value of *m* because the number of data points that one needs in order to gather representative statistics on the permutations grows with the length of those permutations. If, in expectation, one wants 100 chances for each of the m! possible permutations to be discovered in a given window, there must be at least 100m! data points in that calculation. However, even this minimum window size can be problematic in practice, leading to high variances in the probability estimates from Equations (2) and (4). For our purposes, this is a real issue: if the chosen value of *W* is too low, the high variances can eradicate interesting features in the PE and WPE curves; too-large *W* values will reduce the resolution of the analysis, thereby washing out short-time-scale anomalies. To work around this, it is often helpful to increase *W* until the results stabilize, and that is the approach taken here. Since this will depend on the underlying statistics of the signal, careful PE and WPE calculations require some case-by-case hand-tuning and/or testing. (This is true of many other data analysis methods, of course, though that is not widely appreciated in many scientific fields.)

Unfortunately, the literature offers little rigorous advice regarding how to choose *m*; the general recommendation is 3≤m≤6, without any formal justification. The convergence proofs mentioned in the first paragraph of this section require that m→∞; that would require an infinitely long time series (viz., W≥100m!) and is obviously unreasonable for real-world data. In practice, this choice is a balance between detail and data length: short permutations cannot capture the richness of the dynamics, but long permutations require long calculation windows. If m=2, for instance, then the only dynamics that are captured are “up” and “down.” In a time series with even moderate complexity, the probabilities of these two events are usually roughly similar, so both H(2,τ) and $H_{w}\left(2,\tau \right)$ saturate near 1 and neither PE nor WPE is informative. The richer variety of permutations offered by longer word lengths can more accurately capture the complexity of the time series, but—as described in the previous paragraph—that will drive up the window size and lower the temporal resolution of the analysis. In the face of this, a useful practical strategy is to vary *m* and observe the effects on the features in the PE and WPE curves. For real-world time series, those features often stabilize at very low *m* (hence the loose recommendation cited in the first sentence of this paragraph). In the case of the data studied here, the features of the PE and WPE curves stabilized at m=3. To be conservative, we used m=4 for all the calculations reported in this paper; this, in turn, dictated a minimum window size of 2400 points. As a further test, we explored a range of *m* and *W* values around these specific choices, confirming that the features in the PE and WPE curves remained the same. There is, of course, no formal guarantee that this tuning procedure will converge to good choices for this key free parameter, but similar “persistence” approaches are used in many different data analysis approaches (e.g., delays and dimensions for delay-coordinate embedding [31] or length scales for topological data analysis [32]).

The third and final free parameter in PE and WPE calculations is the delay, τ. In the literature, it is customary to fix τ=1, and this is precisely what we do in the anomaly detection study reported in this paper. There is also serious traction to be gained by varying the value of this parameter, though, including exploration of the time scales on which different events occur; see [8] for more details.

A final issue here is the timeline. Recall that the *original* δD and $\delta^{18}$O measurements were spaced evenly in depth but unevenly in time. Because timeline deformation will obfuscate the results of permutation entropy calculations, one must transform the measured data to an even timeline, as described in the last paragraph of Section 2.1, before invoking Equation (1) or (5). Linear interpolation, the standard practice in paleoclimate data analysis, can be problematic in this context, as the repeating, predictable patterns introduced by interpolation can skew the distribution of the permutations and thereby lower the PE and WPE values (see Endnote [33]). This effect will generally worsen with depth because more interpolation is required lower in the core, where the temporal spacing between the measured samples is larger. As mentioned in Section 2.1, the uncertainty of the age scale also comes into play here—another effect that worsens with depth [26]. For all of these reasons, one cannot compare WPE values across wide ranges of the timeline of an ice core.

Permutation entropy techniques have been used successfully to detect a variety of changes in time series: e.g., epileptic seizures in EEG signals [34], bifurcations in the transient logistic map [34], voiced sounds in a noisy speech signal [11], and market inefficiencies in financial records [35,36,37]. They have also been used to assess predictability of time series in a variety of fields [13,14] and reveal various interesting effects in paleoclimate data [7,8,38,39]. The bulk of this work used the *weighted* variant of the technique. As we will show in this paper, though, PE and WPE work together in a complementary fashion, detecting different kinds of anomalies in paleoclimate records.

## 3. Results

**Figure 2:**
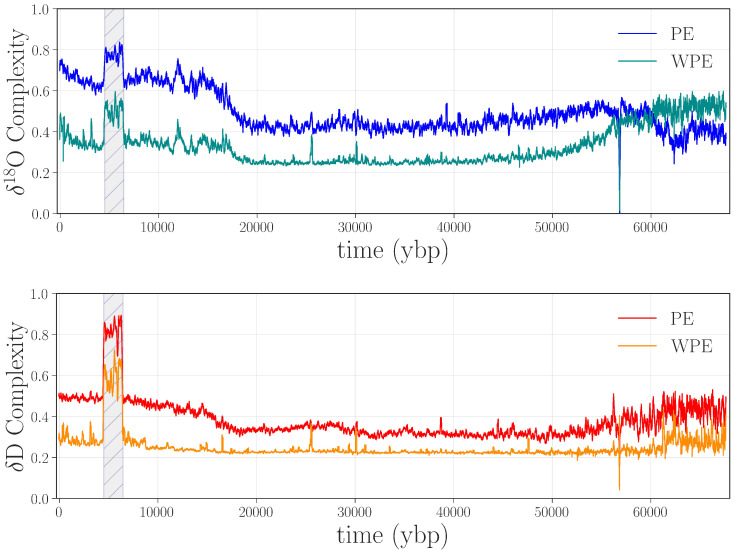
Permutation entropies of the $\delta^{18}$O and δD data from Figure 1, calculated in rolling 2400-point windows (i.e., W=2400) with a word length m=4 and a delay τ=1. **Top panel**: weighted (WPE) and unweighted (PE) permutation entropy of $\delta^{18}$O in cyan and blue, respectively. **Bottom panel**: WPE and PE of δD in orange and red, respectively.

Figure 2 shows the weighted and unweighted permutation entropies of the $\delta^{18}$O and δD data from Figure 1.

There are a number of interesting features in these four curves, many of which have scientific implications, as discussed in [7,8]. Here, we focus on apparent anomalies in the complexity of these climate signals: i.e., discontinuities or abrupt changes in the curves. The most obvious such feature is the large jump in all four traces between ≈4.5 and 6.5 kybp, shown shaded in gray in Figure 2. This sharp increase in the complexity of both signals indicates that something is fundamentally different about this segment of the record, compared with the surrounding regions: in particular, that the values of both δD and $\delta^{18}$O depend less on their previous values during this period than in surrounding regions—i.e., that a large amount of new information is produced at each time step by the system that generated the data. There are two potential culprits for such an increase: data issues or a pair of radical, rapid climate shifts at either end of this time period. Since no such shifts are known or hypothesized by the climate-science community, we conjectured that this jump was due to processing-related issues in the data.

Recall that PE and WPE are designed to bring out different aspects of the information in the data. In view of this, the fact that *both* calculations pick up on this particular feature is meaningful: it suggests that the underlying issue is not just noise—which would be at least partially washed out by the weighting term, causing the jumps in WPE to be smaller than those in PE. Rather, there may be other causes at work here. Going back to the laboratory records and the original papers, we found that an older, less precise, instrument was used to analyze this section of ice, and that this region of the core received the poorest possible quality score [19,40]. This part of the core is from the aforementioned brittle zone, which shatters when removed from the ice sheet. This not only makes the timeline difficult to establish, as mentioned in Section 2.1, but can also affect the δD and $\delta^{18}$O values, as the drilling fluid can penetrate the ice and contaminate the measurements [19]. This could easily disturb the amplitude-encoded information in the core. Unfortunately, permutation entropy techniques cannot tell us what the underlying causes of this anomaly are; that requires expert analysis and laboratory records. Even so, their ability to flag problems and help experts form scientific hypotheses about their causes is a major advantage of these information-theoretic techniques.

**Figure 3:**
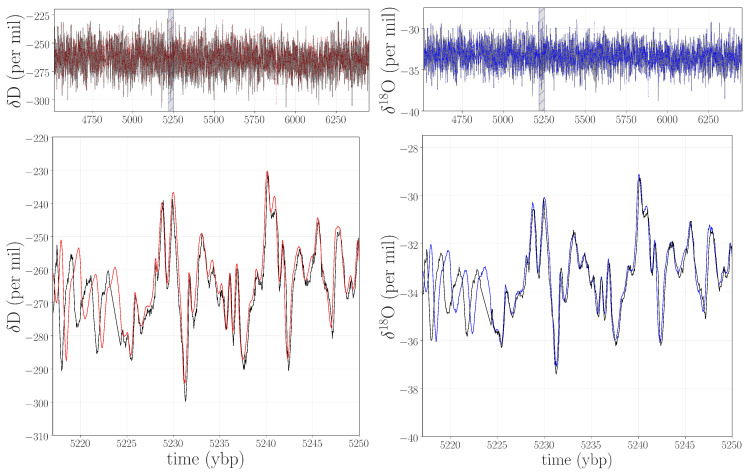
Top panels: the remeasured δD (red) and $\delta^{18}$O (blue) data points in the range of ≈4.5–6.5 kybp, with the original records shown in gray. Bottom panels: a closeup of a small segment of those traces, marked with a gray box in the top panels. Note that the vertical scales here are different than in Figure 1.

As a case in point, we tested our hypotheses about the gray-shaded jump in Figure 2 by remeasuring the 331 m segment of the WAIS Divide ice core that corresponds to this time period. After obtaining the archived ice from the National Science Foundation Ice Core Facility (NSF-ICF), we remeasured the isotope data with state-of-the-art equipment, repeating the depth-to-age conversion and temporal regularization processes described in Section 2. Plots of these new traces appear in Figure 3, with the old values shown in black.

Visual examination of these traces makes two things apparent: lower levels of high-frequency noise in the new signals and small phase offsets between the old and new data. The lower noise levels were, of course, the point of the resampling. The minor phase disparities are a consequence of updates in the laboratory pipeline and the difficulties posed by this particular section of the core. In the years since the creation of the first generation of this system—which was used to measure the original data—there have been a number of updates and improvements. As a result, there are subtle differences (on the order of a few centimeters) in the depth registration between the remeasured and the original data. This is a particular challenge in the brittle zone, since the broken or shattered ice pieces are difficult to piece back together and can settle along fractures, reducing the length of the ice stick by a few centimeters. This can even cause complete data loss in short segments. Remeasuring this ice using state-of-the-art technology has allowed us to improve the data in several important ways, such as by reducing the overall noise levels, improving the depth registration, and even filling in missing parts of the original record that we obtained upon remeasuring the archived ice.

**Figure 4:**
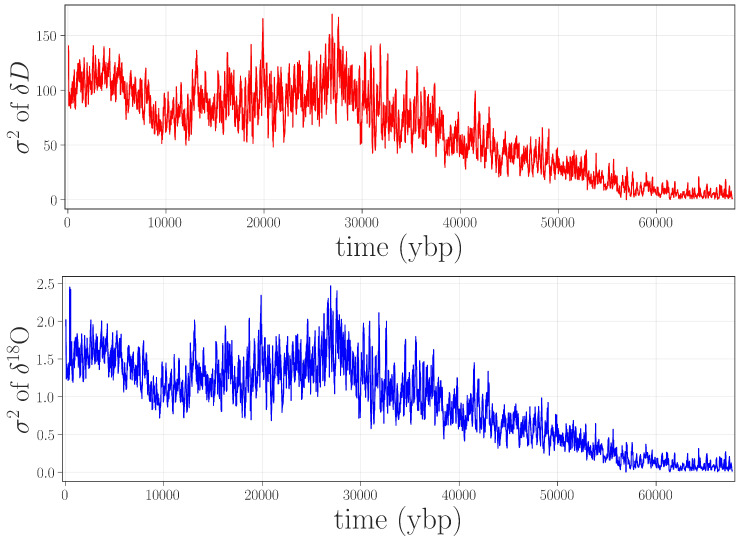
A simple anomaly detection algorithm. Here, $\sigma^{2}$ is estimated on a rolling 2400-point overlapping window for δD (**top**) and $\delta^{18}$O (**bottom**). Neither the feature in the gray-shaded box in Figure 2 nor the other anomalies described later in this paper are brought out by this calculation.

Given the kind of issues that were present in the data, such as increased small-scale variance, it is worth considering whether simpler approaches—less computationally complex than permutation entropy, and with fewer free parameters—would be equally effective in flagging the gray-shaded jump in Figure 2. Because of the inherent data challenges (unknown processes at work on the data, lack of replicates, laboratory issues, etc.), this community has been traditionally limited to fairly rudimentary approaches to anomaly detection: e.g., discarding observations that lie beyond five standard deviations from the mean. Accordingly, we performed a rolling-variance calculation on the same isotope records using a 2400-point window. These results, shown in Figure 4, do not bring out the ≈4.5–6.5 kybp feature, nor do they identify the other anomalies that are described later in this paper.

Of course, there are surely other anomaly detection methods that could be useful for identifying anomalies in paleoclimate records. However, permutation entropy methods identify these problems quite clearly, are relatively simple and fast to compute and, while they have several parameters to tune, the results are quite robust to these choices.

**Figure 5:**
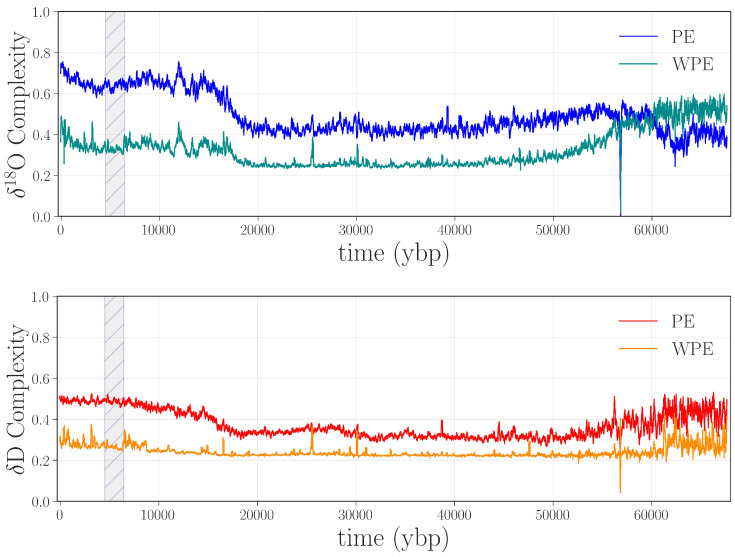
PE and WPE of $\delta^{18}$O (**top panel**) and δD (**bottom panel**) using remeasured data from 1037–1368 m (≈4.5–6.5 kybp).

The last step in validating that the jump in the curves in Figure 2 indicated an anomaly was to replace the data points between 4.5 and 6.5 kybp in the δD and $\delta^{18}$O records of Figure 1 with the remeasured values from Figure 3 and repeat the PE and WPE calculations. The results are quite striking, as shown in Figure 5: the large square waves are completely absent from the PE and WPE traces of the repaired data set.

This experiment—the longest high-resolution remeasurement and reanalysis of an ice core that has been performed to date—not only confirms our conjecture that this anomaly was due to instrument error but also allowed us to improve an important section of the data using a targeted, highly focused effort.

The repaired segment of water-isotope data (≈4.5–6.5 kybp) captures a climate signal from the Holocene era, and thus may contain useful information about the onset of climactic shifts during the beginnings of human civilization. In view of this, recognizing and repairing issues with these data is particularly meaningful. Again, permutation entropy calculations alone cannot tell us the *underlying mechanism* for abrupt changes. However, their ability to identify a region of the core that should be revisited—because of issues that would only have been apparent with a laborious, fine-grained traditional analysis of the data—is a major advantage. This result highlights the main point that we wish to make in this paper: PE and WPE are useful methods for identifying anomalies in time-series data from paleoclimate records. This is particularly useful in contexts where the data are difficult and/or expensive to collect and analyze. In these situations, a tightly focused new study, specifically targeting the offending area using permutation entropy, can maximize the benefit with minimal effort.

Another obvious feature in Figure 5 is the downward spike at around 58 kybp in all four curves. Alerted by this anomaly in WPE and PE, we returned to the laboratory records and found that 1.107 m (110.1 years) of ice was unavailable from the record in this region, and so a span of ≈2387 points in the δD and $\delta^{18}$O traces were filled in by interpolation. This series of points—a linear ramp with positive slope—translated to a long series of “1234” permutations. This causes a drop in the PE curves as the calculation window passes across this expanse of completely predictable values. Indeed, for calculations with τ=1 and W=2400, there is a brief period where 99.45% of the “data” in that window has the same permutation, which causes WPE to fall precipitously, then spike back up as the window starts to move back onto non-interpolated data.

**Figure 6:**
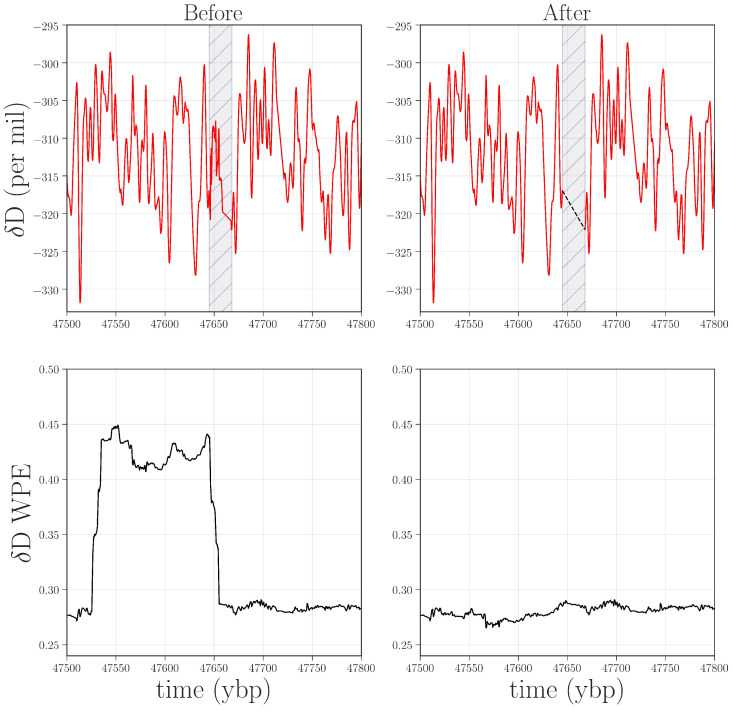
Top row: δD data from 47.5 to 47.8 kybp before and after removal of and interpolation over (black dashed line) a range of faulty values (shaded in gray). Bottom row: WPE calculated from the corresponding traces. The width of the square wave in the lower left plot is the size of the WPE calculation window (2400 points at 1/20th year per point) plus the width of the anomaly. The horizontal shift between the earliest faulty value and the rise in WPE is due to the windowed nature of the WPE calculation.

The large jump from ≈4.5 to 6.5 kybp and the spike at 58 kybp are only a few of the abrupt changes in the permutation entropy traces. The other spikes and dips in those curves, we believe, are also associated with anomalies in the data. Some of these features appear in both PE and WPE; others appear in PE but not WPE (e.g., at 38.7 kybp in δD) or vice versa (e.g., 25.6, 30.1, and 47.5 kybp for δD and $\delta^{18}$O). This brings out the differential nature of these two techniques and the power of the combined analysis: each can, independently, detect types of anomalies that the other misses. In the remainder of this section, we dissect a representative subset of the anomalies in Figure 5 to illustrate this claim, beginning with the case in which WPE, but not PE, suggests data issues. Manual re-examination of the data in the regions around 25.6, 30.1, and 47.5 kybp revealed that the δD and $\delta^{18}$O signal in these regions had been compromised in very small segments of the record (<1 m of ice). The top left panel of Figure 6 shows the region around 47.5 kybp. The signal is visibly different in the small shaded region, combining sharp corners and high-frequency oscillations—unlike the comparatively smooth signal in the regions before and after this segment. WPE is much better at picking up this kind of anomaly because there has been a drastic shift in the information that is encoded in the *amplitude* of the signal. PE, in contrast, does not flag this segment because the distribution of permutations in this region is similar to that of original signal. However, the variance of each delay vector is quite different than in the surrounding signal: an effect to which PE is blind, but that WPE is designed to emphasize.

There are many potential mechanisms that could be causing this distortion in the amplitude: extreme events, for instance, such as large volcanic eruptions, or the same kinds of data and data-processing issues that are discussed above. Returning again to the laboratory records, we discovered that the CRDS-CFA system had malfunctioned while analyzing this region. We could, as before, confirm that this was the cause of the anomaly by remeasuring this region of the core. If the outliers disappeared as a result, that would resolve some significant data issues with only a minimal, targeted amount of effort and expense. If they did *not* disappear, then the permutation entropy calculations would have identified a region where the record warranted further investigation by paleoclimate experts. To date, we have not carried out this experiment, but plan to request archived ice from the NSF-ICF for remeasurement and reanalysis. Access to this limited and irreplaceable resource is, justifiably so, highly guarded—particularly in regard to the deeper ice. In lieu of that experiment, we reprocessed the data from the region surrounding 47.5–47.8 kybp in the δD record by removing the offending data points and interpolating across the interval. As shown in the bottom-right panel of Figure 6, this removes the small square wave from the WPE trace. Without being able to remeasure this ice, of course, we cannot narrow down the cause of this anomaly. Even so, the WPE results are useful in that they allow us to do some targeted reprocessing of the data in order to mitigate the effects.

**Figure 7:**
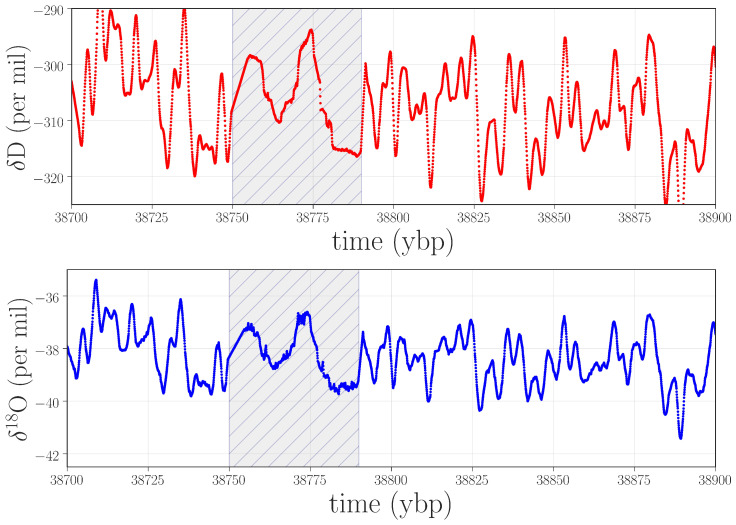
Isotope data near 38.7 kybp that produced a spike in PE but not in WPE. According to the lab records, the graphical user interface froze during the analysis of this segment of the ice, compromising the results.

Spikes in PE that are *not* associated with equally strong spikes in WPE are indications of effects that are dominated by small-scale noise. Figure 7 shows an example of the isotope record in one of these regions.

In contrast to the situation in Figure 6, which is dominated by high-frequency oscillations, this anomaly takes the form of a strong low-frequency component with a small band of superimposed noise. The weighting strategy in WPE causes it to ignore these features, but PE picks up on them very clearly (see Endnote [41]). To diagnose the cause of this anomaly, we again returned to the lab records, finding that the graphical user interface froze while analyzing this particular ice stick, and that the data-processing routines involved in distance registration and isotope content were compromised.

Finally, there is a single spike (near 16.6 kybp in the WPE of δD) where visual inspection of the raw data by expert paleoscientists does not suggest any issues, nor are there any recorded problems in the laboratory records. This is not part of any sections of the core that are known to be problematic, like the brittle zone, and most likely warrants further investigation.

## 4. Discussion

This manuscript illustrates the potential of information theory as a forensic tool for paleoclimate data, identifying regions with anomalous amounts of complexity so that they can be investigated further. As a proof-of-concept demonstration of this claim, we used permutation entropy techniques to isolate issues in water-isotope data from the WAIS Divide core, then remeasured and reanalyzed one of the associated regions using state-of-the-art laboratory equipment. The results of this experiment—the longest segment of replicate ice-core analysis in history—verified that the issue flagged by these information-theoretic techniques in that region were caused by outdated laboratory techniques, as we had conjectured. We also showed that permutation entropy techniques identified several other smaller anomalies throughout the record, which we showed were caused by various minor mishaps in the data processing pipeline. Finally, unlike other anomaly detection studies that use either PE or WPE, we showed that using these two techniques together, rather than in isolation, enables the detection of a much richer landscape of possible anomalies.

This reanalysis approach is already providing valuable insights that reach well beyond the scope of this work. Compared with the prohibitive cost and time commitment of collecting a new core, remeasuring ice from an archived core can be comparatively easy and inexpensive if the region requiring reanalysis can be precisely and clearly defined. This forms the basis for what we believe will be an effective *general* targeted reinvestigation methodology that can be applied not only to the water-isotope record from the WAIS Divide ice core but also to other high-resolution paleoclimate records. Indeed, several such records are being finalized at the time of this publication, which makes this a perfect time to start developing, applying, and evaluating sophisticated anomaly detection methods for these important data sets. Excitingly, as a result of the study reported in this manuscript, PE-based techniques are now being added to the quality control phase of the analysis pipeline for at least one of these new high-resolution records.

The information in paleoclimate records contains valuable clues and insights about the Earth’s past climate, and perhaps about its future. While these records hold a lot of promise, it is crucial—especially in an era of rampant climate-change denial—to be careful when extracting the useful and meaningful information from these records while simultaneously identifying regions that are problematic. For a multitude of reasons, distinguishing useful information from a lack thereof can be a particularly challenging task in paleodata. This is especially true in parts of these records that are hard to analyze, such as the brittle zone of the WDC. Our work has identified a number of intervals in this core where the data require a closer look, including several that contain information corresponding to the time period of the dawn of human civilization.

## Abbreviations

The following abbreviations are used in this manuscript:

CFA	Continuous Flow Analysis
CRDS-CFA	Cavity Ring-Down Spectroscopy Continuous Flow Analysis
NSF-ICF	National Science Foundation Ice Core Facility
PE	Permutation Entropy
WPE	Weighted Permutation Entropy
WAIS	West Antarctic Ice Sheet
WDC	WAIS Divide Ice Core
